# Peri-Operative Morbidity Associated with Radical Cystectomy in a Multicenter Database of Community and Academic Hospitals

**DOI:** 10.1371/journal.pone.0111281

**Published:** 2014-10-31

**Authors:** Luke T. Lavallée, David Schramm, Kelsey Witiuk, Ranjeeta Mallick, Dean Fergusson, Christopher Morash, Ilias Cagiannos, Rodney H. Breau

**Affiliations:** 1 Division of Urology, Department of Surgery, The Ottawa Hospital, University of Ottawa, Ottawa, Ontario, Canada; 2 Clinical Epidemiology Program, Ottawa Hospital Research Institute, Ottawa, Ontario, Canada; 3 Department of Otolaryngology, The Ottawa Hospital, University of Ottawa, Ottawa, Ontario, Canada; University of British Columbia, Canada

## Abstract

**Objective:**

To characterize the frequency and timing of complications following radical cystectomy in a cohort of patients treated at community and academic hospitals.

**Patients and Methods:**

Radical cystectomy patients captured from NSQIP hospitals from January 1 2006 to December 31 2012 were included. Baseline information and complications were abstracted by study surgical clinical reviewers through a validated process of medical record review and direct patient contact. We determined the incidence and timing of each complication and calculated their associations with patient and operative characteristics.

**Results:**

2303 radical cystectomy patients met inclusion criteria. 1115 (48%) patients were over 70 years old and 1819 (79%) were male. Median hospital stay was 8 days (IQR 7–13 days). 1273 (55.3%) patients experienced at least 1 post-operative complication of which 191 (15.6%) occurred after hospital discharge. The most common complication was blood transfusion (n = 875; 38.0%), followed by infectious complications with 218 (9.5%) urinary tract infections, 193 (8.4%) surgical site infections, and 223 (9.7%) sepsis events. 73 (3.2%) patients had fascial dehiscence, 82 (4.0%) developed a deep vein thrombosis, and 67 (2.9%) died. Factors independently associated with the occurrence of any post-operative complication included: age, female gender, ASA class, pre-operative sepsis, COPD, low serum albumin concentration, pre-operative radiotherapy, pre-operative transfusion >4 units, and operative time >6 hours (all p<0.05).

**Conclusion:**

Complications remain common following radical cystectomy and a considerable proportion occur after discharge from hospital. This study identifies risk factors for complications and quality improvement needs.

## Introduction

Bladder cancer is the ninth most common cancer worldwide and represents a significant disease burden with an estimated 72,570 new cases and 15,210 attributed deaths in the United States alone in 2013 [1], [2]. Management of patients with muscle invasive bladder cancer remains challenging since the standard treatment involves removal of the bladder, reproductive organs, pelvic lymph nodes, and creation of a urinary diversion (radical cystectomy) [3]. This extensive resection and reconstruction is associated with considerable peri-operative morbidity to the patient. Common complications of radical cystectomy include significant blood loss, infections, wound complications, venous thrombosis, and metabolic disturbances [4]–[6].

Historical complication rates may not reflect the contemporary patient experience for several reasons. First, new technology and surgical tools are available to reduce blood loss and surgical times [7]–[10]. Second, health care system changes such as pre-operative checklists and hospital care maps have been widely implemented and may improve quality of care and peri-operative outcomes. Finally, surgeon and hospital case volume have been shown to impact radical cystectomy outcomes and a trend toward centralization of radical cystectomy to tertiary care centers has been observed in some regions [11], [12]. For these reasons we felt it was important to evaluate contemporary complication rates to verify if progress has been made.

The American College of Surgeons’ National Surgical Quality Improvement Program (NSQIP) was initiated to supply feedback to participating hospitals by providing accurate information on patient outcomes within 30 days of surgery [13]. NSQIP provides risk-adjusted surgical outcome measures allowing hospitals to identify targets requiring quality improvement [13]. A previous review of cystectomy patients treated from 1991–2001 at 123 NSQIP hospitals reported a 30% incidence of at least one peri-operative complication [5]. Since 2002, NSQIP has grown considerably and now includes over 500 hospitals.

The objective of this study is to characterize complications following radical cystectomy in a contemporary cohort and explore patient and surgical risk factors for adverse events. We hypothesize that despite advancements in peri-operative care, radical cystectomy continues to be associated with a high rate of peri-operative complications. Study results will inform healthcare professionals, facilitate pre-operative patient counseling, aid decision making by identifying vulnerable patients, and identify the most common complications that can be targeted for prophylactic intervention.

## Patients and Methods

Institutional ethics review board approval was obtained from the Ottawa Health Science Network Research Ethics Board. Written informed consent was not obtained as patient information was anonymized and de-identified prior to analysis. De-identified and anonymized data was captured from *Participant Use Data Files* that include patients from hospitals enrolled in The American College of Surgeons’ National Surgical Quality Improvement Program (NSQIP) from 2006 to 2012. NSQIP data is derived from academic and community hospitals, predominantly in North America, and provides highly accurate information including patient demographics, pre-operative comorbidities, and complications within 30 days of surgery. A combination of automated data collection and trained surgical clinical reviewers capture and extract data at each hospital site [13]. Information on outcome is strictly defined and confirmed through medical record reviews and, if necessary, direct patient contact. Frequent data audits are performed revealing an inter-rater reliability of approximately 98% [13]. Eligible procedures are sampled by NSQIP from all participating centers by either capturing all cases at the center or by systematically including cases using a rotating 8-day cycle [13]. NSQIP hospitals are the source of data used in this analysis; however, NSQIP has not reviewed the methodology of this study and is not responsible for its content.

A consecutive cohort of radical cystectomy cases within NSQIP were included using Current Procedural Terminology codes. Patient demographics and medical comorbidities included: age, race, gender, body mass index, American Society of Anesthesiologists’ classification (ASA class), bleeding disorder, pre-operative weight loss, pre-operative sepsis, chronic steroid use, diabetes, dialysis, disseminated cancer, pre-operative chemotherapy, pre-operative radiotherapy (in the last 90 days prior to surgery), dyspnea, alcohol use, smoking history, functional status, chronic obstructive pulmonary disease, congestive heart failure, pre-operative albumin concentration, pre-operative creatinine level, emergency status of cystectomy, >4 units of red blood cell transfusion prior to surgery, operative time, and urinary diversion type. In total 27 characteristics were available for baseline comparisons. Criteria for each characteristic are defined in the 2012 NSQIP User’s Guide [13]. No information about treating institution, surgeon, or tumors (i.e. tumor stage or grade) was available.

The incidence of peri-operative complications within 30 days of surgery was reported. The NSQIP definition for each complication is available in the NSQIP User’s Guide [13]. Complications examined in this analysis included: infectious complications (urinary tract infection, superficial and deep surgical site infection, sepsis), hematologic complications (bleeding [≥1 unit red blood cell transfusion intra-operatively or within 72 hours of surgery]), deep vein thrombosis/thrombophlebitis (DVT), and other complications (wound disruption/fascial dehiscence, acute renal failure, prolonged ventilation, unplanned intubation, cardiac arrest requiring cardiopulmonary resuscitation, myocardial infarction, and death). The severity of individual complications and management required for complications is not collected.

The primary study outcome was the occurrence of any peri-operative complication. Associations between individual patient and surgical factors with the occurrence of any complication was determined using log binomial regression to directly estimate relative risks with 95% confidence intervals. A multivariable log binomial regression analysis was then performed to adjust for confounding by incorporating variables that were statistically significant on univariable analysis and those that have been associated with cystectomy complications in previous studies.

Variables with >15% missing data (low serum albumin concentration, pre-operative radiotherapy, pre-operative chemotherapy) were added to the baseline multivariable model to determine adjusted associations with post-operative complications in an exploratory analysis. For all analyses no adjustment was made for multiple testing and a p-value ≤0.05 was considered statistically significant. SAS software version 9.4 for Windows was used for analyses (Cary, NC, USA).

## Results

From 2006 to 2012, 2303 radical cystectomy patients were captured by NSQIP. Patient and surgery characteristics are presented in Table 1. Of note, 1115 (48%) patients were 70 years old or older, 1911 (85%) were White, and 1819 (79%) were male. The median body mass index was 28 (IQR 24 to 32). Median hospital stay was 8 days (IQR 7 to 13 days).

**Table 1:** Baseline patient and surgical characteristics of radical cystectomy cases in NSQIP from 2006–2012.

Variable	n (%)
Age (years)	Mean 67.9 (SD 11.2)
<65	800 (34.7)
65–70	388 (16.9)
70–75	400 (17.4)
>75	715 (31.1)
Race	
White	1911 (84.8)
Other	115 (5.1)
Missing	227 (11.2)
Gender	
Female	482 (20.9)
Male	1819 (79.0)
BMI	Mean 28.4 (SD 5.9)
<25	671 (29.4)
25–<30	836 (36.6)
30–<35	511 (22.4)
≥35	265 (11.6)
ASA class	
1–2	611 (26.5)
3–5	1690 (73.4)
Bleeding disorder	94 (4.1)
Pre-operative weight loss (>10% over 6 months)	77 (3.3)
Pre-operative sepsis	14 (0.6)
Steroid use (chronic)	71 (3.1)
Diabetes	446 (19.4)
Dialysis	16 (0.7)
Disseminated cancer	114 (5.0)
Chemotherapy (≤30days before surgery)	
Yes	137 (6.0)
Missing	1024 (44.5)
Radiotherapy (≤90 days pre-operatively)	
Yes	11 (0.5)
Missing	1024 (44.5)
Dyspnea	241 (10.5)
Alcohol use (>2 drinks/day)	
Yes	56 (2.4)
Missing	1019 (44.3)
Current smoke	571 (24.8)
Smoking history	
≤50 pack years	852 (37)
>50 pack years	150 (6.5)
Missing	1301 (56.5)
Functional status	
Independent	2230 (96.8)
Dependent	70 (3.0)
Chronic obstructive pulmonary disease (severe)	191 (8.3)
Congestive heart failure (≤30 days pre-operatively)	19 (0.8)
Pre-operative albumin concentration (g/dl)	Mean 3.9 (SD 0.6)
>4.1	488 (21.2)
3.8−4.1	341 (14.8)
3.5−3.7	261 (11.3)
<3.5	308 (13.4)
Missing	905 (39.3)
Pre-operative creatinine level (mg/dl)	Mean 1.17 (0.7)
≤1.3	1689 (73.3)
>1.3–<2	426 (185)
≥2	113 (4.9)
Missing	75 (3.3)
Emergency cystectomy	9 (0.4)
>4 units RBC transfusion (≤72 hours pre-operatively)	57 (2.48)
**Surgical characteristics**	
Operative time	
≤6 hours	1364 (59.5)
>6 hours	927 (40.5)
Continent urinary diversion	442 (19.2)

*Missing data not shown for variables where <2% of patients had missing data.*

BMI = body mass index.

ASA class = American Society of Anesthesiologists’ classification.

NSQIP = National Surgical Quality Improvement Program.

RBC = red blood cells.

### Incidence and timing of complications

Overall, 1273 (55.3%) patients experienced at least 1 post-operative complication by the 30^th^ post-operative day. The most common complication was blood transfusion within 72 hours of surgery (n = 875; 38.0%). If transfusion is not included as a complication, 659 (28.6%) experienced at least 1 post-operative complication by day 30. Infectious complications were common with 218 (9.5%) urinary tract infections, 193 (8.4%) surgical site infections, and 223 (9.7%) sepsis events. Seventy-three (3.2%) patients had fascial wound dehiscence. Eighty-two patients (4.0%) developed a DVT, 22 (1.0%) patients had cardiac arrest, and 67 (2.9%) patients died.

**Figure 1:**
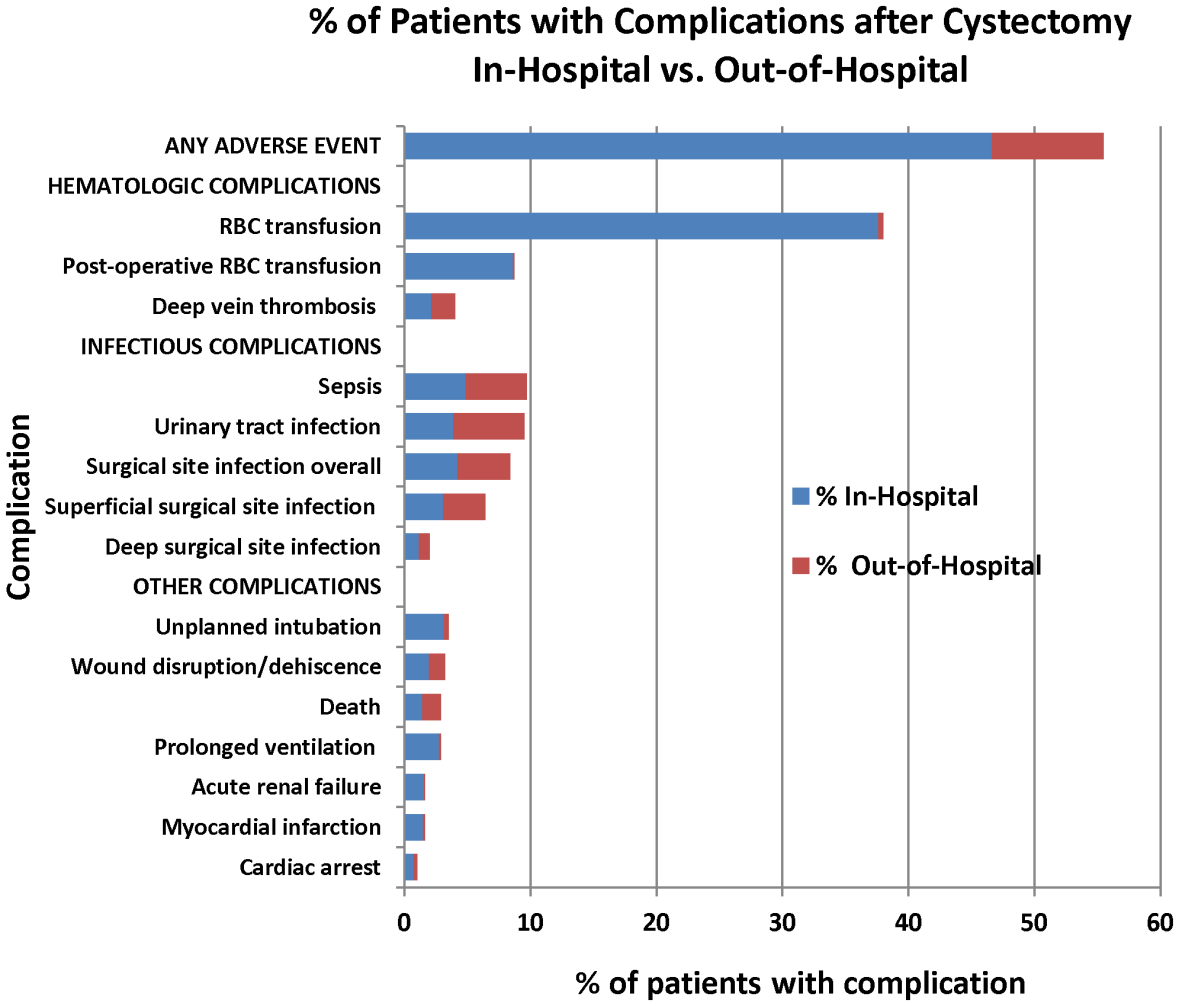
Percentage of patients who received radical cystectomy in NSQIP from 2006–2012 and experienced complications within 30 days of surgery. Complications are presented by type and by in-hospital versus out-of-hospital diagnosis. RBC = Red blood cell.

One hundred and ninety-one (15.6%) complications occurred after discharge from hospital including: 127 (58.8%) urinary tract infections, 37 (46.3%) DVTs, 28 (39.4%) wound dehiscences, and 25 (50.0%) deaths (Table 2 and Figure 1).

**Table 2:** Frequency of complications after radical cystectomy in NSQIP from 2006–2012.

Variable	n (%)	Days post cystectomyMedian (IQR)	n (%) after discharge from hospital
Patients experiencing at least 1 complication	1273 (55.3%)	Mean 4.3 (SD 7.1)	191 (15.6)
**Hematologic complications:**			
Red blood cell transfusion (RBC)		Mean 0.6 (SD 2.3)	
Day of surgery (including intra-operative)	875 (38.0)		N/A
Within 72 hours post-operative	200 (8.7)		
Deep vein thrombosis	82 (4.0)	14 (8.5–20)	37 (46.3)
**Infectious complications:**			
Sepsis	223 (9.7)	12 (8–18)	111 (50.2)
Urinary tract infection	218 (9.5)	15 (9–21)	127 (58.8)
Surgical site infection overall	193 (8.4)		94 (50.3)
Superficial surgical site infection	147 (6.4)	13 (8–18)	75 (52.5)
Deep surgical site infection	46 (2.0)	12 (7–17)	19 (43.2)
**Other complications:**			
Death	67 (2.9)	14.5 (9–21)	25 (50.0)
Prolonged ventilation (>48 hours)	67 (2.9)	6 (3–11)	3 (4.6)
Unplanned intubation	80 (3.5)	6 (2–12)	9 (11.4)
Wound disruption/fascial dehiscence	73 (3.2)	11.5 (7.5–16)	28 (39.4)
Cardiac arrest requiring CPR	22 (1.0)	8 (5–15)	5 (25.0)
Myocardial infarction	37 (1.6)	3 (1.5–5.5)	2 (5.6)
Acute renal failure	36 (1.6)	8 (3.5–16.5)	6 (16.7)

Complications are presented by type. The absolute number, percentage, and occurrence of complications in-hospital versus out-of-hospital are shown.

CPR = cardiopulmonary resuscitation.

N/A = not available.

### Associations between patient and surgical factors with the occurrence of at least one complication

On univariable analysis, several factors were significantly associated with an increased risk of at least one post-operative complication (primary outcome). This included female gender, high ASA classification (ASA 3–5), dependent functional status, chronic obstructive pulmonary disease, pre-operative weight loss, pre-operative dyspnea, pre-operative transfusion, and operative time >6 hours (Table 3). Many of these factors maintained clinical and statistical significance on multivariable analysis (Table 4).

**Table 3:** Univariable analysis of patient and surgical factors with complications following radical cystectomy in NSQIP.

Variable	Relative Risk	95% Confidence Interval	p-value
**Patient factors**			
Age (Increase of 1 year)	1.00	1.00–1.01	0.01
Non Caucasian race	1.11	0.95–1.29	0.18
Female gender	1.19	1.10–1.29	<0.0001
Body mass index (≥35 vs <25)	1.08	0.96–1.21	0.19
ASA class 3–5	1.17	1.07–1.28	0.0006
Bleeding disorder	1.06	0.89–1.26	0.5
Pre-operative weight loss	1.21	1.02–1.42	0.02
Pre-operative sepsis	1.39	1.16–1.66	0.0004
Steroid use	1.10	0.91–1.33	0.33
Diabetes	1.01	0.92–1.11	0.79
Dialysis	1.13	0.77–1.66	0.52
Disseminated cancer	0.98	0.83–1.17	0.85
Pre-operative chemotherapy	1.09	0.93–1.28	0.28
Pre-operative radiotherapy	1.40	0.97–2.01	0.07
Dyspnea	1.15	1.04–1.28	0.009
Alcohol	0.89	0.67–1.18	0.41
Current smoker	1.00	0.92–1.08	0.93
Dependent functional status	1.12	0.92–1.35	0.26
COPD	1.15	1.03–1.30	0.02
History of TIA	0.92	0.60–1.40	0.70
Congestive heart failure	0.95	0.62–1.46	0.82
Decrease in pre-operative albumin	1.19	1.12–1.27	<0.0001
Increase in pre-operative creatinine	1.02	0.98–1.06	0.33
Pre-operative transfusion >4 units	1.34	1.15–1.58	0.0003
Emergency case	1.01	0.56–1.81	0.99
**Operative factors**			
Operative time greater than 6 hours	1.26	1.17–1.35	<0.0001
Continent urinary diversion	0.94	0.86–1.04	0.24

ASA class = American Society of Anesthesiologists’ classification.

COPD = chronic obstructive pulmonary disease.

TIA = transient ischemic attack.

**Table 4:** Multivariable analysis of patient and surgical factors with complications following radical cystectomy in NSQIP.

Variable	Relative Risk	95% Confidence Interval	p-value
Age (Increase of 1 year)	1.01	1.00–1.01	0.004
Female gender	1.19	1.09–1.29	<0.0001
Current smoker	0.99	0.91–1.09	0.87
Body mass index (≥35 vs <25)	1.08	0.96–1.22	0.21
ASA class 3–5	1.13	1.03–1.25	0.01
Pre-operative weight loss	1.15	0.98–1.35	0.09
Pre-operative sepsis	1.23	1.01–1.49	0.04
Dependent functional status	1.02	0.83–1.25	0.87
COPD	1.14	1.02–1.29	0.02
Increase in pre-operative creatinine	1.02	0.96–1.09	0.50
Diabetes	0.98	0.89–1.08	0.65
Pre-operative transfusion >4 units	1.28	1.07–1.54	0.006
**Operative factors**			
Operative time greater than 6 hours	1.30	1.20–1.40	<0.0001
Continent urinary diversion	0.99	0.89–1.09	0.79

*Multivariable analysis had a sample size of 2183.*

ASA class = American Society of Anesthesiologists’ classification.

COPD = chronic obstructive pulmonary diseaseReferences.

Variables with >15% missing data (low serum albumin concentration [39% missing], pre-operative radiotherapy [44% missing], pre-operative chemotherapy [44% missing]) were added to the baseline model to determine their adjusted associations with post-operative complications. Low serum albumin concentration (RR 1.16 95%CI 1.06–1.26, p = 0.0006) and pre-operative radiotherapy (RR 1.54 95%CI 1.13–2.09, p = 0.006) were independently associated with experiencing one or more complication.

## Discussion

Accurate characterization of peri-operative morbidity facilitates patient counseling and identifies targets for quality improvement interventions. We studied a large historical cohort of patients treated from 2006 to 2012 at hospitals participating in The American College of Surgeons’ National Surgical Quality Improvement Program (NSQIP) [13]. Two results of this study are particularly noteworthy: first, the incidence of complications after radical cystectomy remains high with 55.3% of patients experiencing at least one post-operative complication by 30 days. Second, a significant proportion (15.6%) of complications occurred after discharge from hospital, including 46.3% of DVTs.

Findings from this study are consistent with previous reports from multi-institutional cystectomy databases and large single center cohorts in which complication rates of 20–60% by 30 days were reported [3]–[6], [14]. The most common complications in this study were: red blood cell transfusion (44.7%), urinary tract infections (9.5%), surgical site infections (8.4%), and sepsis (9.7%). The incidence of each of these complications is slightly higher than reported in a previous review of the NSQIP hospitals from 1991–2002 and is in the higher range compared to other similar studies [5], [15]. These differences may be due to differences in the study populations, as the earlier NSQIP cohorts were comprised of Veterans Affairs Medical Centers with only 1% of the sample being female, compared to 21% of our cohort. Consistent with previous studies, female gender is associated with complications (RR 1.18 95%CI 1.09–1.28), possibly due to vaginal entry and dissection [15], [16]. A second reason that may explain a higher complication rate in this cohort is the rigor of complication assessment, which is a particular strength of NSQIP.

The timing of complications in this study has implications for physicians as a significant proportion (15.6%) occurred after discharge from hospital, most notably infectious complications, DVTs, and fascial dehiscence. A previous study of Surveillance, Epidemiology, and End Results (SEER)-Medicare data showed that as many as one quarter of patients require readmission after cystectomy with 33.8% of those having complications [17]. This high rate of out-of hospital complications should prompt physicians to examine ways to monitor these patients for complications using early follow-up visits/testing and to improve methods for preventing complications after discharge.

Despite ongoing efforts to improve radical cystectomy peri-operative care including refinement of surgical technique, technological advances, and thrombotic/infectious prophylaxis, complications remain high and improvement is needed. Improved outcomes begin with proper patient selection. This study and others have identified factors that are associated with poor outcomes such as: increased age, female gender, comorbid conditions, previous radio/chemotherapy, low albumin concentration, and longer duration of surgery. Many risk factors cannot be modified, however knowledge of a high risk patient can aid the treating team to modify their approach and ensure proper patient counselling and monitoring. Some interventions have been shown to decrease surgical complications including: electrosurgical heat-sealing devices that reduce bleeding, pre-operative checklists, surgical care pathways, and adherence to antibiotic and deep vein thrombosis prophylaxis guidelines [7]–[9], [15], [18]. Physicians need to be aware of beneficial interventions at their disposal and ensure they are applied consistently in all cystectomy patients given their high risk of adverse outcomes.

This study has several strengths worth noting. First, compared to large single institution series, the results of this study are more generalizable because NSQIP currently includes data from 547 community and academic hospitals. Data from high volume centers of excellence are of value, however, they are not likely representative of outcomes for patient populations treated at a majority of hospitals [6], [11], [12]. Second, data used for analyses are likely valid. NSQIP collects data prospectively, using trained surgical clinical reviewers. They use pre-defined outcomes that have been shown to be highly accurate [13]. Finally, the large number of procedures included in NSQIP each year makes it a powerful resource for measuring current practice outcomes compared to single institution series spanning several decades over which therapies and health care systems change [3].

NSQIP is a valuable resource; however, inherent restrictions of its data content limit this study’s results and conclusions. Some pre-operative variables that are important to physicians treating bladder cancer are not available such as tumor stage and previous non-surgical treatments. Furthermore, data for some pre-operative variables, such as serum albumin concentration, and pre-operative chemotherapy or radiotherapy treatment have missing data, possibly limiting our ability to detect or characterize important risk factors for adverse events. Finally, we performed multiple tests of significance in our analysis to explore a range of independent variables; this may have allowed for some chance findings.

## Conclusion

In summary, complications after radical cystectomy remain high with 55.3% of patients experiencing at least 1 complication within 30 days of surgery. Several randomized clinical trials are ongoing that attempt to reduce peri-operative complications in cystectomy patients with interventions such as: minimally invasive robotic surgery, intra-operative tranexamic acid to reduce blood loss, and pre-operative nutritional supplementation [19]. This study provides accurate information for pre-operative patient counseling and identifies targets for quality improvement interventions. Physicians performing radical cystectomy should be aware of risk factors, common complications, prevention strategies, and treatment of all complications to ensure optimal patient outcomes.
